# Cranial Conundrum: An Unusual Case of an Epidermoid Cyst in the Prepontine Cistern

**DOI:** 10.7759/cureus.51549

**Published:** 2024-01-02

**Authors:** Joseph Russo, Bradley Thompson, Nida Ansari, Malina Mohtadi, Sacide S Ozgur, Patrick Michael

**Affiliations:** 1 Internal Medicine, St. Joseph’s University Medical Center, Paterson, USA

**Keywords:** cp angle, cerebello-pontine angle tumour, ct and mri brain, cystic brain tumor, transphenoidal surgery, epidermoid cysts

## Abstract

We present a fascinating case of a patient who suffered from persistent headaches for three months due to an epidermoid cyst located in the prepontine cistern. Epidermoid cysts are a very uncommon type of intracranial tumor, known for their slow growth and gradual onset of neurological symptoms. In this particular case, our patient, a 35-year-old, experienced a headache that was accompanied by dizziness, photophobia, and pain when moving their eyes. Further imaging revealed a cystic lesion in the prepontine cistern, which had a mass effect on the pons. After confirming the lesion was likely an epidermoid cyst through an MRI, the patient underwent surgery to have it removed. We hope to highlight the rarity of this type of tumor and its unique features when viewed through imaging.

## Introduction

Epidermoid cysts have an incidence rate of only 1%-2% when considering all intracranial tumors [1]. These growths can originate from ectodermal cells that have migrated to the dorsal midline between the third and fifth weeks of embryonic development, coinciding with the closure of the neural tube [2]. French pathologist Cruveilhier described them as the “most beautiful tumors of all the tumors” because of their pearly consistency [1]. Typically, epidermoid cysts are located in the parasellar region, or cerebellopontine angle (CPA), and those in or around the brain stem are deemed rare [1]. Our case highlights the unusual location of an epidermoid cyst in a 35-year-old woman.

## Case presentation

The patient is a 35-year-old Hispanic female with a medical history of recurrent bilateral breast abscesses who presented to the ED with headaches and dizziness that had been ongoing for the past three months. She stated that the headaches previously occurred intermittently, with an insidious onset of episodes of dizziness and pain that occurred mainly in the afternoon and lasted for about three hours. The headache was described as dull, throbbing, and primarily in the forehead and behind her eyes. There was an associated photophobia, blurring of vision, and pain with ocular movements, and the pain was somewhat alleviated by taking ibuprofen or acetaminophen. The dizziness was unchanged with position. She also noted bilateral numbness and tingling in her arms and shoulders. Prior to presentation, for 48 hours, her headache became constant, with a pain severity rating of 8/10. Ibuprofen and acetaminophen were no longer providing relief, prompting her visit to the emergency department. She also noted an unintentional weight loss of 4 kg and night sweats over the past three months. She denied any recent upper respiratory illness or sick contacts. She denied any syncope, fall, loss of balance, fever, or weakness. On the physical exam, the patient was alert and in no acute distress. Her strength and sensation to light touch were intact throughout her upper and lower extremities. She had mild hearing loss in the left ear with no other focal neurologic deficits.

**Figure 1:**
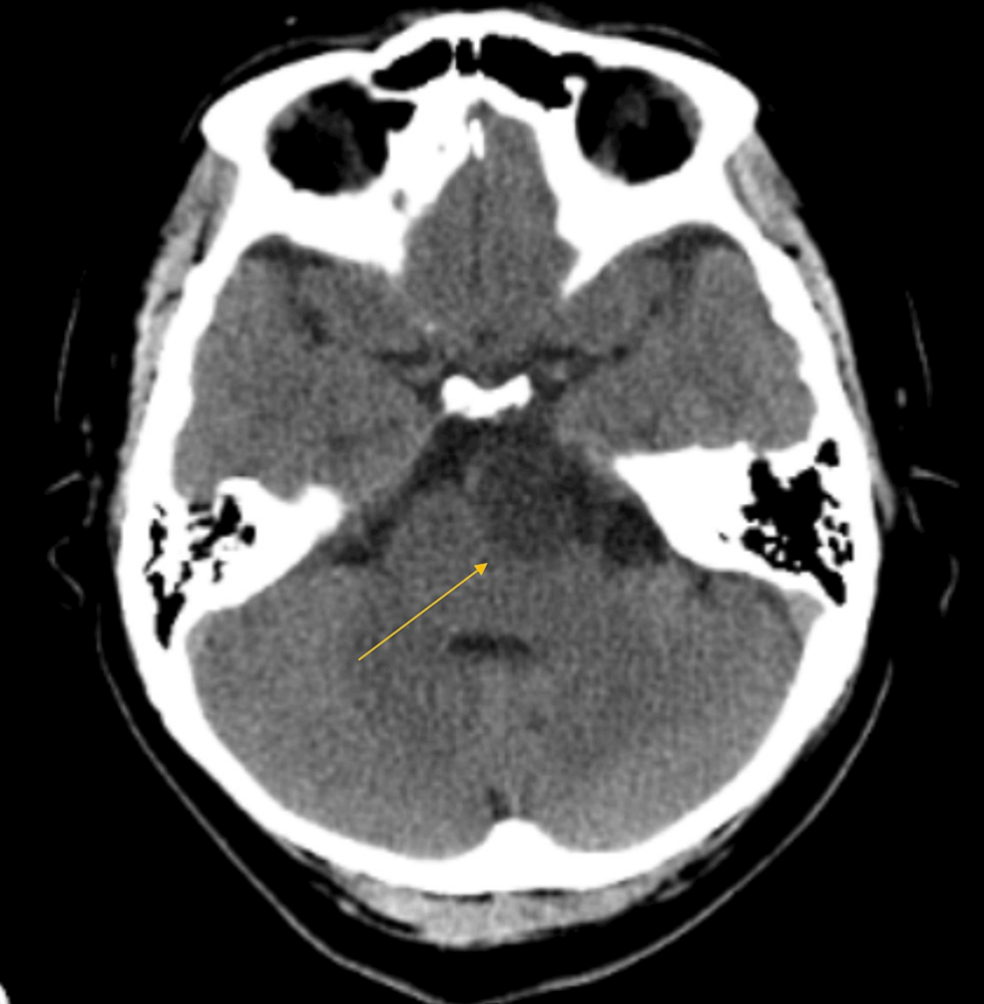
A non-contrast CT scan of the head demonstrates a cystic lesion in the left prepontine cistern with a mass effect on the left pons.

In the ED, she was normotensive without tachycardia, saturating at 99% on room air, and afebrile. Laboratory studies did not reveal any abnormalities. A CT scan without contrast of the head showed a cystic lesion in the left prepontine cistern with a mass effect on the left pons (Figure 1).

**Figure 2:**
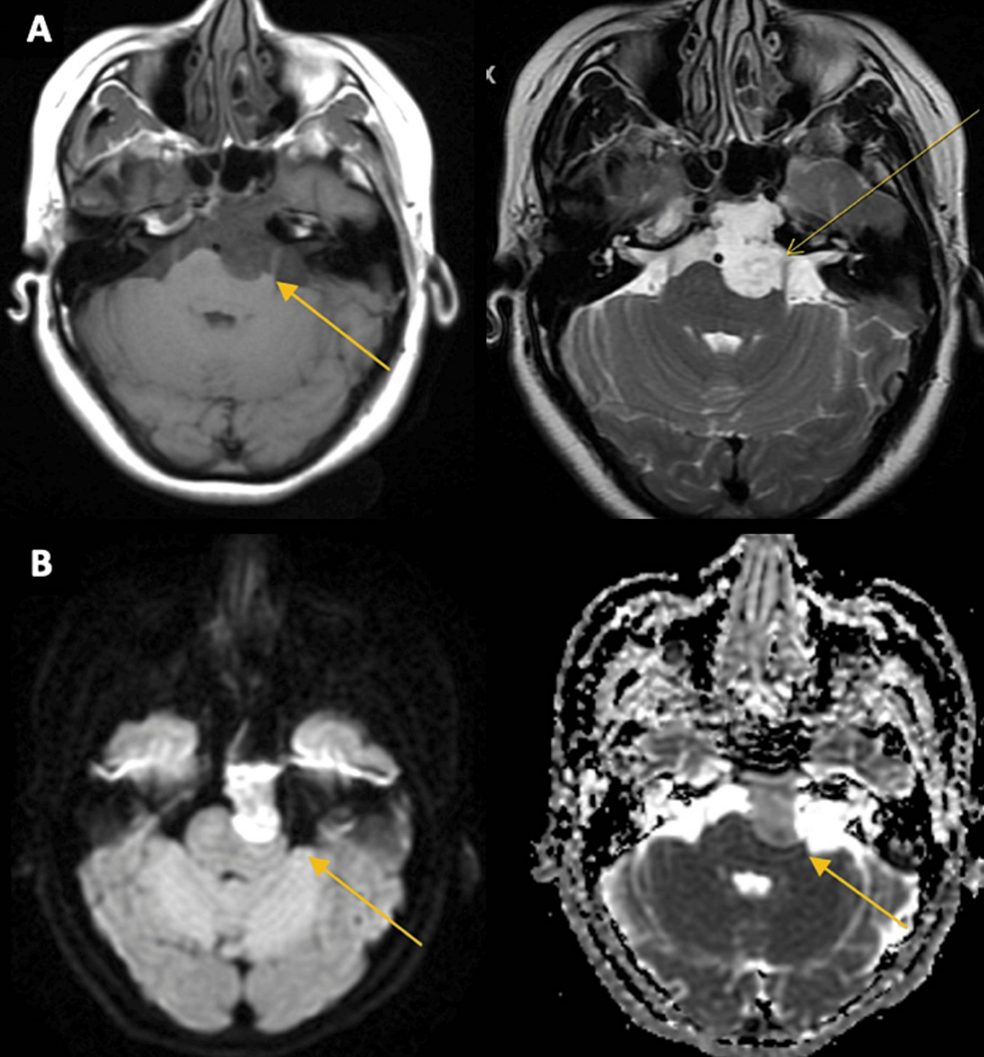
An MRI of the brain demonstrates a lesion of the prepontine cistern with a mass effect on the left pons and fourth ventricle, with restricted diffusion and small amounts of internal enhancement. A: T1 imaging, pre-contrast, and post-contrast B: diffusion-weighted imaging and diffusion coefficient imaging

The patient was started on IV dexamethasone. Subsequent MRI imaging of the brain with and without contrast showed a lesion of the prepontine cistern with a mass effect on the left pons with restricted diffusion and small amounts of internal enhancement measuring 3.1 cm x 1.7 cm, suspicious for an epidermoid cyst (Figure 2).

**Figure 3:**
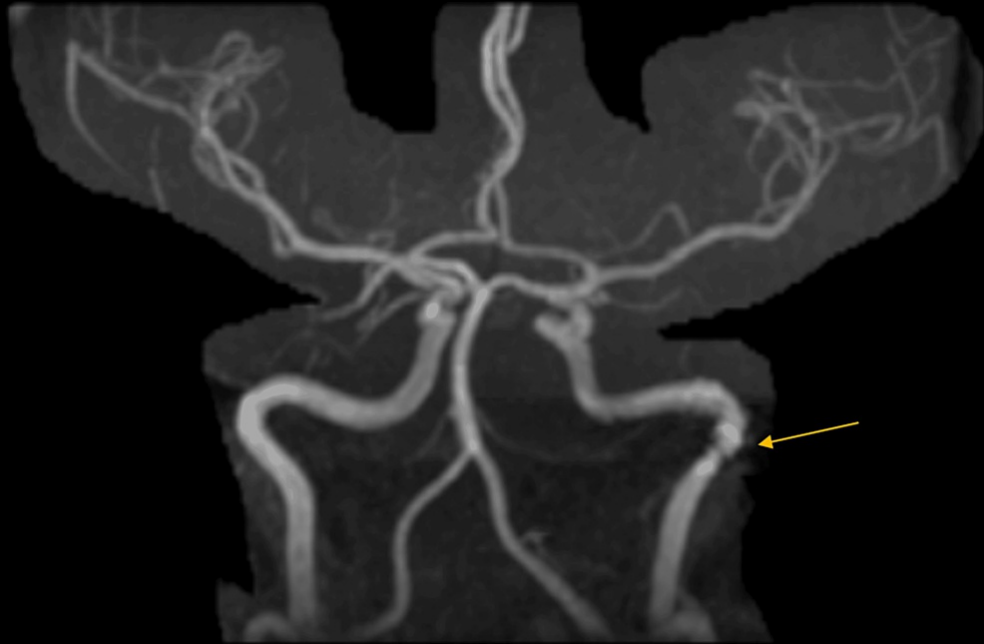
Magnetic resonance angiography projection imaging shows an irregularity within the left internal carotid artery near the carotid canal with a slight loss of flow.

Magnetic resonance angiography (MRA) showed irregularity within the left internal carotid artery near the carotid canal with a slight loss of flow (Figure 3).

**Figure 4:**
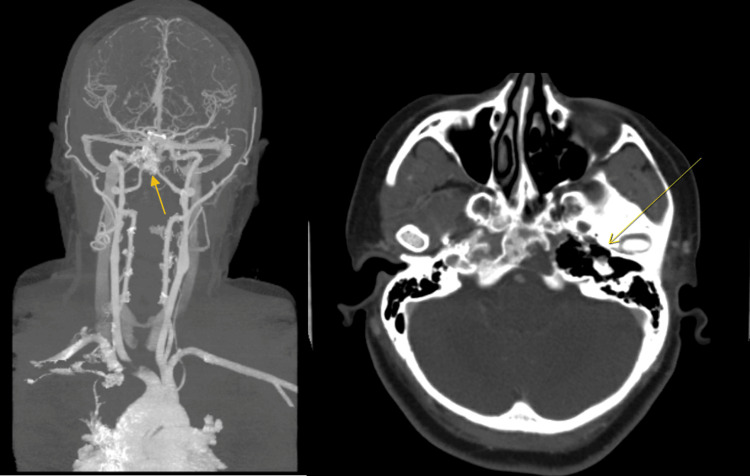
A CT angiogram-processed image shows that the epidermoid cyst at the skull base did not cause a gross invasion or occlusion of the left internal carotid artery. Subsequent imaging demonstrated bone loss at the petrous apex and adjacent clivus.

A follow-up CT angiogram (CTA) showed the epidermoid cyst at the skull base, causing bone loss of the petrous apex and adjacent clivus extending to the medial margin of the internal carotid artery within the foramen lacerum, without gross invasion or occlusion of the vessel (Figure 4). 

The patient was admitted for further management, and neurology was consulted. Her headache remained persistent throughout her stay but was better controlled with pain medication. Neurosurgery was consulted for possible intervention. An MRI of the spine was done to evaluate for other cyst formations or drop mets from a different pathology; however, it did not show any evidence of enhancing lesions. Infectious disease was also consulted to rule out infectious etiology; however, it was concluded that there was a low likelihood of an infectious process. A lumbar puncture could not safely be performed due to concern for increased intracranial pressure. Neurosurgery concluded that the patient would benefit from surgical resection; however, due to equipment limitations at our institution, it was not feasible, so the patient was transferred to a neighboring hospital for transsphenoidal resection of the cystic lesion. A CT scan without contrast of the head was done two months post-operation, as the patient reported to the ED with a chief complaint of headache. The scan demonstrated resection of the clivus and sphenoid sinuses.

## Discussion

Epidermoid cysts are incredibly slow-growing tumors whose growth pattern mirrors epidermal skin cells [1]. The emergence of neurological symptoms generally occurs gradually [3]. They have a peak incidence in the fourth decade of life, with the most common presenting symptom being headache [2, 4]. Additional symptoms include hemiparesis, impairment of the seventh cranial nerve, dysfunction of the sixth cranial nerve, and unsteadiness in gait [1]. The growth of the tumor is due to the accumulation of desquamated epithelial cells, leading to a buildup of keratin and cholesterol and producing a milky white, pearly appearance [1]. Roughly 60% of all intracranial epidermoids are typically found within the CPA [2, 4]. In terms of CPA tumors, they rank as the third most frequent type, following acoustic neuromas and meningiomas [2]. The second most common location within the posterior fossa is the fourth ventricle, which comprises approximately 5%-18% of all intracranial epidermoids [2]. About 15% of epidermoids occur within the middle cranial fossa, while instances of epidermoids appearing in the spinal region are exceptionally rare [2,4,5]. As for imaging, a key diagnostic indicator is the presence of a mass resembling cerebrospinal fluid (CSF) that infiltrates cisterns, usually enveloping nearby neurovascular structures. Epidermoids typically exhibit a hypointense appearance on T1-weighted MRI scans, with the possibility of displaying intermediate intensity between the brain and CSF on T1. Additionally, they tend to appear hyperintense on T2-weighted sequences [3]. However, the signal intensity may vary, contingent on the relative proportions of lipids, cholesterol, keratin, and proteins, and they rarely exhibit contrast enhancement [3].

In our case, an epidermoid cyst was discovered in a rather peculiar place, at the left prepontine cistern, impinging on the left anterior aspect of the pons. These so-called “beautiful” tumors tend to grow and encapsulate vessels and nerves [2, 6]. In our case, on the CTA of the brain and head, the cyst appeared to extend to the medial margin of the internal carotid artery within the foramen lacerum, accounting for the slight irregularity noted on the previous MRA of the head and brain. Luckily for the patient, no gross invasion or occlusion of this vessel was appreciated. The patient's cyst was resected without complications via transphenoidal resection due to its peculiar location. A CT scan without contrast of the head was performed two months after the surgery, as the patient reported to the ED with a chief complaint of headache. The scan demonstrated resection of the clivus and sphenoid sinuses. According to the radiographic report, it was noted that there was soft tissue in the sphenoid sinuses, which could have represented either packing material or mucosal thickening. No recurrence of the cyst was noted. No postoperative complications have since been reported. Favorable long-term results with minimal morbidity have been attained through a more cautious strategy in terms of surgery when dealing with challenging cases [1]. Radical resection will prevent recurrence but is not always possible as the capsule tends to adhere to neurovascular structures [1].

## Conclusions

This report presents a rare and intriguing case of an epidermoid cyst in the left prepontine cistern, demonstrating the complexities associated with diagnosing and treating such unusual intracranial tumors. Characterized by slow growth and the insidious onset of symptoms like headache, dizziness, and photophobia, epidermoid cysts require careful evaluation. The CT and MRI studies were pivotal in revealing the cyst's unique position and impact on surrounding structures, including the left internal carotid artery. Despite the challenges posed by the cyst's location and the potential risks of surgery, the patient underwent successful transsphenoidal resection at a specialized facility, avoiding complications such as gross invasion or occlusion of the internal carotid artery. Successful surgical removal at a specialized center underscores the importance of expert surgical skills and interdisciplinary collaboration. This case highlights the need for precise diagnosis and treatment strategies to manage uncommon neurological disorders and contributes to the broader understanding of epidermoid cysts in atypical cranial locations.
